# Fourth International Dental Congress, St. Louis, Mo., August 29 to September 3, 1904

**Published:** 1904-11

**Authors:** 


### FOURTH INTERNATIONAL DENTAL CONGRESS, ST. LOUIS, MO., AUGUST 29 TO SEPTEMBER 3, 1904.

*Opening Session—Monday, August 29, 1904.*

(Continued from page 810.)

Dr. A. W. Harlan, of New York, called upon by the President to introduce the foreign delegates to the Congress, made a few appropriate remarks in presenting Dr. Otto Zsigmondy, Vienna, the official representative of Austria, who spoke as follows:

Mr. President, Ladies, and Gentlemen,—I thank you from the bottom of my heart for this very kind and enthusiastic reception. In Austria, as in other countries, a remarkable change has taken place, in the sense that dentistry is now fully recognized by the government as being of the utmost importance to public hygiene. As a proof of the increasing interest our authorities are taking in dental matters, I should state that, not content to gather information about international dental congresses from the official reports, they deemed it fit to appoint an official delegate to this Congress. The desire often expressed by the Austrian dental societies may now be considered almost realized.

I wish to be permitted to mention another improvement which is likewise the result of our efforts. Special dental courses have been organized for army surgeons in which they can obtain not only the special training desirable, but at the same time they attend gratuitously to the teeth of the soldiers of the Vienna garrison.

To these statements I have only to add my sincerest thanks for the privilege of having been permitted to address to you these words, and to express a confident hope that the results of this great and memorable Congress will bear further good fruit for my country also. (Applause.)

Dr. Rudolph Weiser, of Vienna, Austria, delegate from the Central Union of Austrian Stomatologists and the Association of Dentists of Prague, was next introduced and spoke as follows:

Mr. President, Ladies, and Gentlemen,—If the wonderland America is in general a voyage-aim for the cultivated classes of society, a dental congress in the United States will exert upon us as professionals an attractive power to a most exceptional extent.

The Americans have always been ahead in converting theoretical results into practical applications of benefit to mankind. Consequently, and by natural logic, they ought to distinguish themselves in dentistry, and indeed they may be considered pioneers in this branch of knowledge.

I should now like to inform you that we Austrians have a still greater incentive to study your institutions thoroughly and to enter into friendly personal contact with you because the universities in our country are just about to place education in this branch of surgery upon a new and sound basis.

I have the honor to express to you, in the name of the Central Union of Austrian Stomatologists and of the Association of the Czech Dentists of Prague, the most cordial thanks for your kind invitation and warm reception. As the delegate of the aforenamed dental associations I feel happy to transmit you the sincerest wish that the results of your enterprise may be of real benefit to the nations of the world. (Applause.)

Dr. Harlan next introduced Dr. Alfred Burne, the representative of Australia, who spoke as follows:

MR. PRESIDENT, LADIES, AND GENTLEMEN,—I trust I shall be pardoned this morning if I take for my cue a line from the ghost in Hamlet, "Brief let me be." I feel that there are so many to follow me that I do not desire to take your time. From the state of Victoria, New South Wales, I desire to extend to the Congress congratulations and good wishes for a very successful meeting. I feel that we who have come such long distances to this Congress are like the wise men who followed the star of Bethlehem. We are looking to the Fourth International Dental Congress, seeking the light that we need, and which I think we shall find. Gentlemen, I thank you. (Applause.)

Dr. J. M. Magee, of St. Johns, N. B., Canada, was next introduced and spoke as follows:

MR. PRESIDENT, LADIES, AND GENTLEMEN,—I came here simply as a dentist, and it was with the greatest distress that I learned I would be called upon to speak for the great country to the north. I am very sorry that I cannot respond on behalf of the profession in Canada as befits the occasion, but the representative who could worthily represent it is unfortunately absent, and I was unexpectedly called upon to take his place. I am very proud to be honored on this occasion by being permitted to speak to you, and, as my predecessor from the antipodes expressed it, I thank you. (Applause.)

Dr. E. Sauvez, of Paris, was next introduced. He said, Mr. President, in the name of the government of the French Republic, of which I have been commissioned delegate together with my eminent *confrère* and friend Dr. Godon, I have the honor of saluting the aurora of the Fourth International Dental Congress.

I will also present you the good wishes for the success of this meeting on behalf of the Directeur de l'Assistance Publique of Paris, who has delegated us for the purpose; and finally, as president of the French committee on publicity and propaganda and of the national federation of France, I want to offer you the assurances of our sentiments of confraternity and sincere admiration for the constant efforts which American dentists have been making for so many years for the purpose of elevating the status of our profession.

The École Dentaire, Paris, which represents the most important institution for dental education and the most vital one in our country, takes the greatest interest in your work, in your journals, in your schools, because you have travelled towards the ideal with gigantic strides. Sixty-five years ago the first dental school in the world was created in Baltimore. To-day about sixty-five schools are being operated upon United States territory. It must be clear to every one that a country capable of creating on an average a school per year must necessarily be the one in which the status of the profession is the highest.

We are particularly happy to bring to you our token of admiration in this beautiful city of St. Louis, the heart of the United States, where so many names remind us of France, the first one to give to the modern centuries, through the cession of Louisiana, the example of a colony voluntarily ceded by the parent country, and when we look at the prosperity of your beautiful country, and at the splendor and importance of your Exposition, we cannot refrain from thinking of the just statements made by Livingston, the ambassador of the United States, at the time of the signing of the Louisiana Purchase. He then prophesied the future in such a precise way that you have not hesitated to inscribe part of it upon the monument commemorating the event.

Dr. W. D. Miller, Berlin, the representative from Germany, was greeted with loud applause.

*Dr. Harlan.*—It is not necessary, it seems, that I should introduce Professor Miller, our distinguished representative from Germany and the guardian of American dentistry in Europe.

*Dr. Miller.*—Mr. President, ladies, and gentlemen, I do not need to say that I thank you very heartily for this courteous show of friendship on your part, and that I reciprocate it most fully.

I come to you as the representative of the National Dental Association of Germany, of the National Association of Dental Societies, and of the National Association of Dental Faculties of Germany. These three bodies embrace, I may say, practically all the prominent dentists of the country, and they have asked me to convey to you an expression of cordial good-will and sympathy. They wish me to assure you also of their great appreciation of the grand work done in America by the dental profession, and of their hope and confidence that this meeting will, like all other dental meetings held on American soil, contribute much to the advancement of our profession. (Applause.)

*Dr. Harlan.*—I have pleasure in introducing to you the representative of the government of Holland, Dr. John E. Grevers, who was a member of the International Medical Congress held in Washington in 1887, and of the World's Columbian Congress of 1893 in Chicago.

*Dr. John E. Grevers,* Amsterdam, Holland.—Mr. President, ladies, and gentlemen, I thank you very heartily for your kind reception, and I thank you for the opportunity you have given me of coming before you as the representative of a very small country —a country, however, which is full of love and interest for everything pertaining to dentistry. My gracious little Queen Wilhelmina has kindly delegated me to represent her government at this Congress; and this act marks the beginning of a great period for Holland, as the government is becoming more and more interested in matters relating to dentistry. Dentistry in our country has for many years been regulated by law, but not to the best interests of dentistry, I am sorry to say. I think I can now prophesy that the present ministry will do much towards bettering the conditions for those desiring to enter upon the study of our specialty.

I have also come to bring you greetings from two dental societies, the Odontological Society of Holland and the Union Society of Dentists. Both have delegated me to represent them, and to bring to you their heartfelt greetings, and the hope that the outcome of this Congress will be beneficial to dental science throughout the world. Again I thank you for your very kind reception. (Applause.)

Dr. Vincenzo Guerini, of Naples, the representative of Italy, was introduced by Dr. Harlan, and spoke as follows:

Mr. President, Ladies, and Gentlemen,—As the representative of the Odontological Society of Italy and of the Neapolitan Medical Association, I have the honor of bringing to you a fraternal greeting from the Italian dentists.

A strong current of sympathy, originating from great historical events that are known to all of us, unites and will ever unite Italy to America. Every new manifestation of progress, every fresh success of yours, has our sincere applause; and my colleagues in Italy hope that this great dental Congress may add new glory to the many accomplishments which your nation can already boast.

It is the source of great happiness to me to find myself for the first time in the United States, in this great centre from which the light of civilization and of progress radiates so abundantly over the whole world; and this day, when I have the honor of standing in the midst of so eminent an assemblage of talent, constituted for the most part of the elect of the dental profession in America, will ever be one of my dearest remembrances.

In conclusion, gentlemen and ladies, I will express my fervent desire that the brilliant results of this great international congress may constitute a new and solemn affirmation of the autonomy of dental science, which Americans have the merit of being the first to recognize, and which is the indispensable condition of rapid progress in dentistry.

May this Congress strengthen still more the bond that unites all of the dentists of the world into one body, rendering it more easy for the dental profession to achieve fresh triumphs in the field of scientific and practical dentistry for the welfare of humanity. (Applause.)

*Dr. José J. Rojo*, Mexico City, Mexico.—Mr. President, ladies, and gentlemen, I thank you for your kind reception. It is with great pleasure that I have the honor of addressing you as delegate from the National Mexican Dental College and from the Mexican Dental Society. Permit me to express our feelings of appreciation and special sympathy with the organizers of and co-operators in this great historical event, the Fourth International Dental Congress. Besides this, in the name of the dental profession of the republic of Mexico, I want to express our best wishes for a successful meeting, and many beneficent results to our profession. (Applause.)

Dr. Jaime D. Losada, of Madrid, one of the representatives from Spain, spoke as follows:

Mr. President, Ladies, and Gentlemen,—I feel a deep sense of emotion on this occasion, as it is for me a great honor and pleasure to address you in the name of Spain. My government takes the greatest interest in all matters relating to dental science, and as a further proof of this interest has sent, besides myself, as governmental delegates, two of the greatest dental men we have in Spain, Drs. Florestan Aguilar and Dr. Luis Subirana, and it is through their courtesy and also in their names that I address you.

I have to thank you most heartily for the kind way in which we have been welcomed by the city of St. Louis and by the Fourth International Dental Congress. We are full of admiration for your magnificent exhibition, one of the greatest wonders the world has ever seen. This sentiment of admiration is still greater to us, coming as we do from countries which have existed almost since the prehistoric periods, as we realize that your wonderful land has accomplished in a few years—thanks to the progressive genius of the American people—that for which other countries have consumed centuries.

I am happy to say that dentistry is one of the up-to-date sciences in Spain. Our teaching of this branch compares very favorably with that of any other country, and our requirements are very high. My *confrères* in Spain are most progressive, and, of course, as in every part of the world, American systems are in vogue. Many of the young dentists from my country, knowing that America is the true spring of modern dental science and the mother of dental education, come to your land, regardless of sacrifices, with the object of improving their knowledge, and then return to the mother country usually with the American D.D.S. appended to their names.

Again I thank you for your cordial welcome, and my only regret is that I am not familiar enough with your language to be able to express well enough the sentiments of admiration and friendship from my professional brethren in Spain. (Applause.)

Dr. L. C. Bryan, of Basel, the representative of Switzerland, spoke as follows:

Mr. President, Ladies, and Gentlemen,—Considering the late hour, the small spot which I represent on the map of the world, and the large number of speakers to be heard from, I will only extend to you the cordial greetings of the profession of Switzerland, and say that it is our great desire that the next dental congress may be held in our hospitable country, the playground of the world. I thank you very much. (Applause.)

*Dr. Harlan.*—Among those who have been appointed as official delegates to this Congress you have standing before you Dr. J. Y. Crawford, of Nashville, Tennessee, delegate of the United States government. (Applause.)

*Dr. J. Y. Crawford,* Nashville, Tenn.—Mr. President, dental diplomats of the world, ladies, and gentlemen, in all the experiences of my life I have never felt more profoundly my responsibility than in the duty which I am now trying to perform. I extend to you, the representatives of the nations of the earth, on behalf of the government of the United States, our greetings, and welcome you most heartily on this auspicious occasion. The people of America, and particularly the departments of our government, whether it be the executive, legislative, or judicial, are in sympathy with the idea of gathering together at stated intervals congresses representing the various interests of the world, and we come with our congratulations, most earnestly invoking such interest and solicitude in the discussions of odontological questions at this time as will bring great benefits to the world, and result not only in the improvement of the masticating apparatus of the human family, but tend to relieve suffering humanity and add to the comfort of the population of the world. We are created upon the fundamental principle of equal rights to all, and special privileges to none. We therefore enter most heartily into the business of this Congress for the improvement and betterment of the condition of mankind. By the authority of the great government located at Washington, resting upon the will of the people of this great nation, we extend our most hearty congratulations. Mr. President, as the humble representative of the government of the United States, I pledge you to do all that we can for the advancement of the interests of this Congress and the profession of dentistry the world over. (Applause.)

Dr. Edwin P. Tignor, representative of the Army Dental Corps, was next introduced, and spoke as follows:

Mr. President, Ladies, and Gentlemen,—It gives me great pleasure to be able to be present at this great meeting. It was a great surprise to me, and a very agreeable one, to receive the order directing me to proceed here in order to represent the Army Dental Corps, and I thank you very much for the privilege of being allowed to speak to you to-day.

Dr. Harlan next introduced Dr. J. M. Whitney, of Honolulu, the representative of the Hawaiian Islands, who spoke as follows:

Mr. President, Ladies, and Gentlemen,—As I was sitting greatly enjoying the proceedings, my friend Dr. Kirk touched me on the shoulder and said, "We must hear from the baby of our republic," to which I replied, "No, you had better excuse me," but Dr. Harlan says no excuses are acceptable here. I am here not as representing the baby country, because we had an organization in Hawaii long before California was known to most of us. I was privileged to stand before you at the Second International Dental Congress, at that time as a foreigner, but now I am proud to stand before you as one of you. You are one with us and we are one with you, and I thank you for this honor. (Applause.)

*Dr. Harlan.*—Ladies and gentlemen, it affords me particular pleasure to be able to now present to you my old associate and collaborator in the preparation of the proceedings of the World's Columbian Dental Congress of 1893, Dr. Louis Ottofy, the representative of the Philippine Islands. (Applause.)

*Dr. Ottofy.*—Mr. President, ladies, and gentlemen, it is with the greatest of pleasure that I join my distinguished predecessors upon this floor in expressing to you my sincere thanks for your most cordial welcome. I assure you that I highly appreciate the opportunity and privilege of being present to witness the erection of another milestone in the history of the progress of dentistry, a progress which may not inaptly be likened unto the progress of our great nation—a nation which during the last few years has extended her civilizing hand across the seas to a people emerging from the darkness of semi-ignorance into the bright rays of intelligence. Some idea of this influence may be formed when I mention the fact that, while I come from a territory over which wave the Stars and Stripes, I have travelled a greater distance to come here, excepting one gentleman from Java, I believe, than any other member of this Congress.

While here with you, it shall be my sacred duty to learn all I possibly can of the advance made by our profession, and to carry the fruits of your thought and labor back to the Philippines. On behalf of the dental profession of the Philippine Islands, which I have the honor to represent, I again thank you heartily for your cordial welcome. (Applause.)

Dr. Salvador Pratto was introduced by Dr. Harlan, and spoke as follows:

Ladies and Gentlemen,—As delegate of the Odontological Society of Uruguay I desire to express to you our most cordial sentiments of confraternity and esteem.

You no doubt know that there is a little country in the southern extremity of this hemisphere, a small country in area, but large in brains and activity, and its organization in educational lines places it at the head of the South American intellectual movement. In my country, as in all other South American republics, we carefully follow the progress achieved by dentistry in this country, without any delay save that implied by the distance which separates the two countries.

I feel a deep sense of gratification, realizing that I shall be the medium of conveying to the Odontological Society of Uruguay an account of your deliberations and a description of your operative ability and of your discoveries in the field of practical dentistry.

I am a sincere admirer of the genius of America's sons, whom I regard as the first to establish permanently the separate profession of dentistry upon an autonomous basis—thus cultivating the fruits of a plant which germinated in France, and which is now being diffused throughout the universe for the welfare of suffering humanity.

I wish to express my earnest desire for the success of this gathering, and I furthermore wish to say that I feel proud and happy to be a member of the foreign delegation.

*Dr. Harlan.*—In every country with a population of five hundred thousand or over will be found installed somewhere a representative of America practising dentistry. I have pleasure in presenting to you Dr. J. W. Noble, of Hong Kong, China.

*Dr. Noble.*—Ladies and gentlemen, it gives me great pleasure to appear before you this morning, and to have the opportunity to thank you for the cordial reception you have given to the members from foreign countries—with whom I feel I am included this morning—as well as to myself. I regret very much that all those practising dentistry in my part of the world cannot be with me here to-day to see the progress taking place in our profession in science and art. They are missing that which we are gaining, and we can only sympathize with those who are absent from us during the present week. (Applause.)

Dr. Benjamin Vidaurre, representative from Nicaragua, was next introduced, and spoke as follows:

Mr. President, Ladies, and Gentlemen,—The government of Nicaragua, whose duly accredited representative I am proud to be, sends greetings to the Fourth International Dental Congress. Please accept from my profession and myself sincere thanks in the name of my country for the courtesies that have been extended to me. I am glad to say that my government has always shown not only a desire, but an eagerness, to assist in any scientific work or lend aid in any way that will further the ends of progress. Our president, on being invited, did not hesitate to send a representative of the government to the Congress, and while that representative may not shed much light upon the deliberations of this body, he will endeavor to take back with him part at least of the great treasures which will emanate from this Congress. Again I thank you most heartily for the cordial reception tendered me. (Applause.)

Dr. Joaquin Yela, Jr., official delegate of the government and dental faculty of the republic of Guatemala, Central America, was presented, and spoke as follows:

Mr. Chairman, Fellow Delegates, Ladies, and Gentlemen,—If I were to speak now in my own native tongue, very few of you, I am sure, would understand me; although, from my speaking in my broken English the number of sufferers may be greater. Relying then, on your own well-known patience and goodness, I will try to make myself understood in the beautiful language of Shakespeare and Longfellow.

For many reasons I could have wished that it had fallen to the lot of a delegate of greater distinction and experience than myself to respond to your very kind invitation to speak, but I do recognize that in letting your choice fall upon me you have acted, not out of regard to the special delegate, but out of compliment to the country whose government and dental faculty I have the honor, however unworthy, to represent at this Congress; a country which is linked to the United States of America by so many ties both of a commercial nature and of sincere friendship.

I observe wth great pleasure that there are to be discussions and conferences on many and very important dental topics which it will be for us to report to our respective governments and faculties.

It only remains for me now, Mr. Chairman and fellow-delegates, to express once more my very cordial thankfulness for the benevolent reception accorded me here, and to present as well the same sentiment of thankfulness to the people of this beautiful city of St. Louis, assuring you all that I am the interpreter of the feelings of the government, faculty, and people whom I have the honor of representing in this highly distinguished assembly. (Applause.)

Dr. Ragnwald Hendricsen, of Christiania, the representative from Norway, was presented, and spoke as follows:

Mr. President and Members of the Fourth International Dental Congress,—On behalf of the dental profession of Norway and the Christiania Dental Society, it is with the greatest pleasure that I greet you. Norway is the farthest northern country represented here, and although cool of climate we are warm of heart, and our profession as a whole and individually are profound admirers of American dental science. We all try to follow your methods and are eager to know more and more, and therefore have sent and are sending to your colleges many students.

The cordial, friendly spirit which you have shown to all of us foreigners will always be a delightful remembrance, and I wish to express the most sincere thanks both from myself and my Norwegian brethren.

The official representative of the Norwegian government, Mr. Anderson, has not arrived, and I could not refrain from expressing a few words on behalf of my nation. (Applause.)

Dr. N. S. Jenkins, of Dresden, Germany, was presented to the Congress, and spoke as follows:

Mr. President, Ladies, and Gentlemen,—With all my heart I wish to bring to you the greetings of the body of colleagues practising their profession under the American degree in the empire of Germany. They send to you their most cordial and fraternal greetings, and beg you to believe that they are heart and soul with you in every effort towards uplifting and carrying forward the ever-increasing influence of the great profession to which we all belong. (Applause.)

*The President.*—Ladies and gentlemen, members of the Fourth International Dental Congress, the next order of business is the address by the President of the Fourth International Dental Congress, and as an evidence that I never really intended to preside over this body, until the election had been finally ratified on this floor, I am here to-day at this wedding-feast without a wedding-gown,—namely, the "President's address." My remarks therefore shall be brief.

At the threshold of this great gathering of the profession from all parts of the world, I bring you the greeting of the Hon. Theodore Roosevelt, President of the United States, for a most pleasant, profitable, and successful Congress. I desire also to acknowledge the courtesy and interest of his Excellency the governor of Missouri.

To the official delegates representing foreign governments, and the representatives of dental societies in other countries, assembled here to-day, I extend a most cordial and hearty greeting, and beg to express the hope that their stay among us may be most agreeable and pleasant. It is a matter of sincere congratulation to welcome this splendid representation from abroad, and to be able on behalf of the profession in America to felicitate them upon their achievements in the advancement of professional interests in the various countries from which they are accredited.

We in America are pleased to note the great interest which our brethren from abroad evince in this Congress, not only by their presence, but more particularly by their contributions to the excellent programme which has been prepared for this meeting. We welcome them with open hearts, and cordially reciprocate the warm sentiments which they have ever expressed to representatives of the profession from America on occasions like the present.

It is indeed a pleasure to commend the enterprise, enthusiasm, and liberality displayed by State and local societies and colleges in America, in their very generous contributions to assist in defraying the necessary expenditures in preparing for this Congress.

The Organization Committee also desires to acknowledge at this time their indebtedness to the Hon. Howard J. Rogers, Director of Congresses, for the ready and willing support which he has at all times rendered, and for his unfailing interest and solicitude in everything having for its object the complete success of the Congress.

To the members of the profession in America assembled here to-day I bring the greeting of the authorities of the Louisiana Purchase Exposition, and that of the officers and committees in charge of the preparations for this great meeting. In point of numbers this is the largest dental congress ever held, which must be a source of great satisfaction to all our people. The programme, from a literary and a scientific stand-point, has never been equalled. No such array of talent has ever before been brought together, and I am sure that the results achieved at this meeting will make an epoch in dentistry that will for many years be referred to as one of the most brilliant in the annals of the profession.

Many questions will come up for discussion, the correct solution of which will require earnest and thoughtful deliberation, and I urge upon you the necessity of cultivating a broad and liberal spirit in the consideration of the great problems with which you are confronted.

This is not the time nor the place to burden you with references to matters of history or to treat in an academic way the great questions of the hour.

You are conversant with the early history of dentistry, the lack of sympathy and support by the medical profession, the great obstacles to proper professional recognition; the difficulties encountered by the early practitioners; the wonderful achievements with crude facilities for practice, and withal the splendid and brilliant results obtained by the great men who have shed undying lustre upon the profession which they honored and served so well. The past is secure; so let us approach the duties of the hour, with the same spirit of self-sacrifice and love for the profession that animated the fathers of dentistry.

I trust that every member of the Congress will do his utmost to promote harmonious action throughout the various committees and sections, so that all shall work together for the complete success of the Congress and the glory of dentistry. (Applause.)

*The President.*—We are signally honored by having with us to-day the president of the International Dental Federation, a distinguished gentleman who is well known to all of you for his sterling qualities, and for the great ability he has shown not only as a practitioner of dentistry, but as an executive officer, and I now have the honor of presenting to you our distinguished *confrère* from France, Dr. Charles Godon, of Paris, who will address you. (Applause.)

*Dr. Godon.*—Mr. President, ladies, and gentlemen, at this, the first meeting of the Fourth International Dental Congress, I should have liked to speak in the name of France, being one of the official delegates of the French government and of the National Federation, to recall to you all the ties of sympathy which unite our two republics. The fraternal relations existing since the time when you achieved your independence under Washington, with Lafayette and Rochambeau, are also of a professional nature, because of our common ancestors, Gardette and Lemaire, who brought over to America the French dentistry of the eighteenth century; and they are finally assured at the present time because of our common ideals of liberty, equality, and fraternity.

I left to my friend and co-delegate, Dr. Sauvez, the honor of speaking in the name of France, because I have to-day another duty, that is to speak at this opening meeting in the name of an association which is above nationalites, since it is a universal association of national dental societies, a permanent international body existing in the interim of international dental congresses; in the name of this great association which you all know now as the great advisory council on dental international relationships.

The F. D. I. has taken a part in the work of this Congress which for a while was surrounded by many difficulties. But now these are all over, and I bring you in the name of the F. D. I. a fraternal greeting. We can but congratulate you upon this first result, and now we turn over to you our powers and all documents relating to the work accomplished since 1900.

The following statement, which is approved by all the members of the executive council of the F. D. I., will give you an idea of the work which has been performed.

To conclude, let me tell you of my satisfaction in seeing that the F. D. I. has found in your great land this hearty hospitality, characteristic of the American dental profession, for which I thank you sincerely. It is with the greatest confidence that I place its destiny in your hands, quite sure that it will continue to grow and to develop, as does everything here pertaining to the progress of science and to the good of humanity.

*The President.*—If there is no objection this report will be accepted and referred to the Committee on Resolutions.

The Secretary-General, Dr. Kirk, read the following cablegram from Dr. John S. Burnett, of Salto, Uruguay:

"DR. H. J. BURKHART, *President Fourth International Dental Congress, St. Louis:*

"Success to the Congress. I am mentally with you.

"JOHN BURNETT."

The next order of business being the report of the Committee on Prize Essays, the President called upon Dr. Truman to present the report of that committee.

Dr. James Truman, chairman of the Committee on Prize Essays, then read the report.

[This report was published in the October number.]

On motion, the report of the Committee on Prize Essays was accepted and adopted.

The Secretary-General then read a communication from the Dental College of Sheffield, England, as follows:

"UNIVERSITY COLLEGE, SHEFFIELD, DENTAL DEPARTMENT.

"At a meeting of the dental practitioners held in Sheffield, on Tuesday, July 19, 1904, it was unanimously decided to send their congratulations and fraternal greetings to the members in meeting assembled at the St. Louis Dental Congress.

"Also: The members of the Sheffield and District Association of Licentiates of Dental Surgery have pleasure in sending their heartiest good wishes to the members of the Congress at St. Louis.

"The members of the above society have delight in announcing that their honorary secretary, Mr. H. James Morris, L.D.S. (Eng.), will attend the Congress and in person express their hearty good wishes for a happy and successful meeting.

"GEO. HENRY LODGE, *President,*
"CHARLES STOKES,
"FRANK MORDAUNT,
"*Council.*
"H. JAMES MORRIS,
"*Treasurer-Secretary.*"

A letter from Mr. W. H. Williamson, president of the British Dental Association, to Dr. M. H. Cryer, as follows, was received too late to be read, having been delayed in transmission:

"Kindly express to the officials of the Congress my great regret at my inability to be present, and at the same time convey the best wishes of the British Dental Association for the success of your great gathering.

"I shall send a cable on the opening day in case this should not reach you in time.

"Yours sincerely,

"W. H. WILLIAMSON."

*The President.*—If there be no objection, the Secretary-General is instructed to reply to all telegrams and letters to the Congress.

There being no other business before the Congress, the President declared the meeting adjourned until Tuesday morning at ten o'clock.

(To be continued.)

